# Needs and current status of generative artificial intelligence use among college students: a cross-sectional survey based in China

**DOI:** 10.3389/fpsyg.2026.1907218

**Published:** 2026-10-14

**Authors:** Rong Zeng, Chuntao Lei, Qian Jiang, Xueqin Gan, Xiaoguo Zhang, Yu Feng, Qin Xu, Yan Huang, Qian Yang

**Affiliations:** 1 School of Nursing, Chengdu Medical College, Chengdu, Sichuan, China; 2 The First Affiliated Hospital of Chengdu Medical College, Chengdu, Sichuan, China; 3 The First Affiliated Hospital of Traditional Chinese Medicine of Chengdu Medical College, Xindu Hospital of Traditional Chinese Medicine, Chengdu, Sichuan, China; 4 West China School of Public Health and West China Fourth Hospital, Sichuan University, Chengdu, Sichuan, China; 5 Luzhou Medical Device Vocational and Technical College, Luzhou, Sichuan, China; 6 Affiliated Hospital Health School, Southwest Medical University, Luzhou, Sichuan, China

**Keywords:** academic resilience, artificial intelligence, generative artificial intelligence, learning engagement, vocational college students

## Abstract

**Objective:**

This study aimed to investigate the current status of Generative Artificial Intelligence (GAI) usage needs among vocational college students from China, explore their relationships with learning engagement and academic resilience, and identify variables associated with GAI usage demand.

**Methods:**

Using convenience sampling, 3,876 students from five vocational colleges in Sichuan Province were enrolled. Surveys were conducted using the Generative Artificial Intelligence Application Status and Usage Demand Scale, the Learning Engagement Scale, and the Academic Resilience Scale.

**Results:**

The average GAI needs score among vocational college students was 58.23 ± 12.77. Correlation analysis revealed positive relationships between GAI needs and both learning engagement and academic resilience (*p* < 0.01). Multiple linear regression analysis revealed that gender, academic year, availability of GAI learning devices at home, level of GAI understanding, enrollment in GAI-related courses, frequency of active GAI use, student-reported frequency of teachers’ use of GAI in teaching, learning engagement, and academic resilience were independently associated with GAI usage demand (all *p* < 0.05). The model explained 24.4% of the variance in GAI usage demand [*R*^2^ = 0.244, adjusted *R*^2^ = 0.241, *F*(14, 3,861) = 88.932, *p* < 0.001].

**Conclusion:**

Vocational college students’ GAI usage demand was slightly above the theoretical midpoint of the scale. Greater GAI usage demand was independently associated with male gender, earlier academic year, better availability of GAI learning devices at home, a higher level of GAI understanding, enrollment in GAI-related courses, more frequent active GAI use, more frequent student-reported teachers’ use of GAI in teaching, higher learning engagement, and greater academic resilience. Because of the cross-sectional design, these findings should be interpreted as associations rather than causal relationships.

## 1 Introduction

Development Plan for New-Generation Artificial Intelligence” (2017) issued by the State Council emphasizes that Artificial Intelligence (AI) has become the core driving force for the development of various industries and must be placed at a national strategic height for comprehensive layout (State Council of the People’s Republic of China, 2017). General Secretary Xi Jinping further emphasized at the 2019 International Conference on Artificial Intelligence and Education: AI has gradually become an important driving force leading the new round of technological revolution and industrial transformation, which is profoundly changing people’s ways of production, life and learning, and promoting human society to enter an intelligent era of human-machine collaboration, cross-border integration, and co-creation and sharing (Central People’s Government of the People’s Republic of China, 2019). This trend brings historic opportunities for higher education while accompanied by various challenges. In January 2025, the Central Committee of the Communist Party of China and the State Council issued the Outline for Building a Leading Country in Education (2024–2035), which explicitly called for the use of artificial intelligence to support educational transformation (Central Committee of the Communist Party of China and State Council Outline for Building a Leading Country in Education (2024–2035), 2025). Against this background, as an emerging branch of AI technology, Generative Artificial Intelligence (GAI) has attracted extensive attention from academic circles at home and abroad due to its characteristics such as multimodal interaction capabilities, creative output of new content, and high adaptability (Lv, 2023). GAI tools represented by ChatGPT, ERNIE Bot, iFLYTEK Spark, and DeepSeek have been widely applied in teaching practice.

Current scholarship on GAI spans macro-level governance and regulatory discussions (Fu, 2025), the integration of technology with teaching models (Yu et al., 2025), and challenges in educational implementation and management (Chen, 2025). At the level of teaching practice and student development, an editorial summarized the potential opportunities and challenges of AI for supporting student engagement in higher education and highlighted the need for further empirical evidence (Nguyen et al., 2024). Research concerning creativity and broader learning outcomes has produced more nuanced findings. Marrone et al. (2022) reported that students generally viewed human creativity as distinct from and superior to AI creativity, whereas Habib et al. (2024) described both supportive and constraining roles of GAI in student creativity. A meta-analysis reported a significant but small overall effect of GAI on students’ learning outcomes, with variation across outcome types and educational contexts (Zhu et al., 2025). A randomized controlled study further showed that the use of GAI in undergraduate programming education improved students’ computational thinking skills, programming self-efficacy, and learning motivation (Yilmaz and Yilmaz, 2023). Case-based research has also discussed how appropriately designed GAI-supported educational activities may contribute to the development of higher-order thinking skills (Guo et al., 2025). An empirical study found that student–GAI interaction was associated with learning achievement through the mediating roles of self-efficacy and cognitive engagement (Liang et al., 2023). However, evidence concerning student–GAI interaction should not be interpreted as establishing a causal pathway from GAI understanding to general learning engagement. In the present study, level of GAI understanding and learning engagement were therefore treated as conceptually distinct variables and examined separately in relation to GAI usage demand. A separate meta-analysis found that GAI use significantly improved college students’ academic achievement, with a medium overall effect size (Sun and Zhou, 2024).

The present study was conceptually informed by the Technology Acceptance Model (TAM), which proposes that perceived usefulness and perceived ease of use shape individuals’ attitudes and behavioral intentions toward adopting a technology (Davis, 1989). In educational settings, students’ acceptance of and interaction with GAI may also be related to their psychological orientation toward learning. Learning engagement reflects students’ sustained cognitive, emotional, and behavioral investment in learning activities (Schaufeli et al., 2002; Fredricks et al., 2004), whereas academic resilience reflects their capacity to adapt to academic difficulties and actively seek resources to maintain effective learning (Martin and Marsh, 2008). Recent empirical studies have shown that students’ interaction with and acceptance of GAI are associated with cognitive engagement, self-efficacy, and overall learning engagement (Liang et al., 2023; Deng et al., 2026). Emerging evidence has also identified a positive association between students’ AI literacy and academic resilience in AI-supported learning environments (Chen et al., 2026). These findings provide a preliminary basis for considering learning engagement and academic resilience as psychological characteristics associated with students’ demand for emerging AI-supported learning resources. However, because the present study did not measure all core TAM constructs, TAM was used as a broad organizing framework rather than as a complete model tested in this study. Accordingly, learning engagement and academic resilience were examined as theoretically relevant psychological correlates of GAI usage demand rather than as causal determinants.

Vocational college students are important users of GAI technologies and are also directly affected by the integration of GAI into higher education. Guiding students to use these tools reasonably, effectively, and critically has therefore become an important challenge for educational institutions (Preiksaitis and Rose, 2023; Rahman and Singh, 2025; Yu and Chen, 2025). However, quantitative evidence concerning vocational college students’ specific demand for GAI and the demographic, educational, technological, and psychological variables associated with such demand remains limited. Accordingly, this exploratory cross-sectional study aimed to describe vocational college students’ GAI application status and usage demand and to examine the associations of GAI usage demand with learning engagement, academic resilience, and other relevant characteristics. The following research questions were addressed:

*RQ1*: What is the level and dimensional profile of GAI application status and usage demand among vocational college students?

*RQ2*: What are the associations of learning engagement and academic resilience with GAI usage demand among vocational college students?

*RQ3*: Which demographic, educational, technology-related, and psychological variables remain independently associated with GAI usage demand after multivariable adjustment?

## 2 Data and methods

### 2.1 Study subjects

This cross-sectional study was conducted from September to October 2025. Convenience sampling was used to select students from 5 vocational colleges in Sichuan Province as the study participants. The study was approved by the Ethics Committee of Chengdu Medical College (Ethics Approval No.: CYLL2024NO.16). Inclusion criteria: (1) Registered full-time students at the participating colleges; (2) Provided informed consent and voluntarily participated in the study. Exclusion criteria: (1) Students who had suspended their studies or withdrawn from school for any reason.

### 2.2 Data collection

Prior to the commencement of the formal survey, the research team obtained approval from the administrative leadership of each participating institution. Following this approval, the team deployed 1 to 2 trained investigators to each institution to conduct on-site data collection. Before administering the survey, the investigators thoroughly explained its purpose and the estimated completion time (approximately 15–25 min) to the participants. Participants aged 18 years or older provided written informed consent themselves, whereas written informed consent for participants aged 17 years was obtained from their parents or legal guardians before participation. Only after ensuring that the participants fully understood the procedures and provided their informed consent was the survey administered. Data were collected using paper-based questionnaires in either face-to-face or group settings to ensure accuracy and completeness. All participants signed a written informed consent form.

### 2.3 Research tools

#### 2.3.1 General information survey questionnaire

The questionnaire was developed by the researchers and consisted of 13 items: gender, age, academic year, major, place of origin, GAI training experience during high school, home GAI learning equipment configuration, level of understanding of GAI, enrollment in GAI-related courses, frequency of exposure to GAI in professional learning, availability of GAI equipment at school, frequency of active GAI use, and frequency of teachers’ use of GAI in teaching. The level of understanding of GAI was assessed using a single self-reported item with five response categories: no understanding, limited understanding, moderate/basic understanding, good understanding, and excellent understanding. This item reflected students’ perceived familiarity with GAI. It was conceptually distinct from learning engagement, which was assessed using the UWES-S and reflected students’ general engagement in learning across the dimensions of vigor, dedication, and absorption. However, this single-item measure potentially overlapped conceptually with the Understanding and Cognition dimension of the GAI usage-demand scale. Therefore, an additional sensitivity analysis was conducted to examine the potential risk of criterion contamination.

#### 2.3.2 Generative artificial intelligence application status and usage demand scale

Developed by Hu et al. (2025), this scale includes 18 items divided into three dimensions: Understanding and Cognition, Skills and Literacy, and Hopes and Expectations. A 5-point Likert scale was adopted, with scores ranging from 1 (“completely inconsistent”) to 5 (“completely consistent”). In the present study, GAI usage demand was operationalized as the summed total score of all 18 items. The possible total score ranges from 18 to 90, with higher total scores indicating greater self-reported GAI usage demand. No categorical cutoff was applied because the scale does not provide validated thresholds for classifying demand levels. In the present sample, Cronbach’s α coefficients were 0.730, 0.895, and 0.944 for the Understanding and Cognition, Skills and Literacy, and Hopes and Expectations dimensions, respectively, and 0.943 for the total scale.

#### 2.3.3 Daily academic resilience scale

The Daily Academic Resilience Scale developed by Guo (2015) was used to assess students’ academic resilience. The scale was originally developed for middle-school students and comprises 15 items across four dimensions: execution ability (Items 1–5), external support (Items 6–9), self-efficacy (Items 10–12), and emotional stability (Items 13–15). It includes four reverse-scored items. Responses are rated on a 5-point Likert scale ranging from 1 (“completely inconsistent”) to 5 (“completely consistent”), with higher scores indicating greater academic resilience. Because the scale was originally developed in a younger student population, its measurement performance in vocational college students may not be identical to that reported in the original population. In the present sample of vocational college students, Cronbach’s α coefficients were 0.777, 0.786, 0.633, and 0.653 for execution ability, external support, self-efficacy, and emotional stability, respectively, and 0.912 for the total scale. Thus, although the total scale showed high internal consistency, the self-efficacy and emotional stability subscales showed comparatively modest internal consistency.

#### 2.3.4 Utrecht work engagement scale-student version (UWES-S)

The UWES-S was originally developed by Schaufeli et al. (2002) and was subsequently translated and validated in Chinese by Fang et al. (2008). It comprises 17 items across three dimensions: vigor, dedication, and absorption. The original scale uses a 7-point frequency response format ranging from 0 to 6. In the present survey, a 5-point response format ranging from 1 to 5 was adopted to maintain consistency with the response formats of the other major self-report instruments included in the same paper-based questionnaire and to reduce switching between different response formats during survey completion. The resulting possible total score ranged from 17 to 85, with higher scores indicating greater learning engagement. Because this response format differed from that of the original UWES-S, its internal consistency and factorial structure were examined in the current sample. Cronbach’s α coefficients were 0.895, 0.890, and 0.907 for vigor, dedication, and absorption, respectively, and 0.955 for the total scale.

### 2.4 Statistical methods

IBM SPSS Statistics version 26.0 was used for statistical analysis. The internal consistency of the three scales and their respective dimensions was assessed using Cronbach’s α. The factorial structure of the modified UWES-S was examined using exploratory factor analysis with principal component extraction and varimax rotation, with the number of factors fixed at three.

Quantitative data conforming to a normal distribution were described as the mean ± standard deviation (x̄ ± s), while categorical data were presented as frequencies and percentages (*n*, %). Simple linear regression, independent-samples *t*-tests, and one-way analysis of variance (ANOVA) were used, as appropriate, to examine the univariate associations between demographic and GAI-related characteristics and GAI usage-demand scores. Effect sizes were reported as Cohen’s *d* for independent-samples *t*-tests and η^2^ for one-way analyses of variance. Pearson correlation analysis was conducted to examine the associations among GAI usage demand, learning engagement, and academic resilience. Variables with *p* < 0.05 in the univariate analyses, together with learning engagement and academic resilience, were entered simultaneously into a multiple linear regression model using the Enter method, resulting in 14 candidate independent variables. The total GAI usage-demand score was specified as the dependent variable. Unstandardized coefficients, standardized coefficients, and 95% confidence intervals were reported. To examine potential criterion contamination, a sensitivity analysis was conducted using a reduced 15-item GAI usage-demand score that excluded the Understanding and Cognition dimension; the regression analysis was repeated using the same 14 independent variables and the Enter method. A two-sided *p* < 0.05 was considered statistically significant. Given the data-driven variable-screening procedure, cross-sectional design, absence of preregistration, and lack of prespecified confirmatory hypotheses, the regression analyses were considered exploratory and were interpreted as associations rather than causal or confirmatory effects. Although participants were recruited from five vocational colleges, participant-level institutional identifiers were not available in the analytic dataset. Therefore, the analyses could not account for the clustering of students within colleges, and the reported standard errors and confidence intervals should be interpreted cautiously.

## 3 Results

### 3.1 General characteristics and univariate analysis

A total of 4,044 questionnaires were distributed in this survey. After excluding 168 invalid questionnaires, 3,876 valid questionnaires were recovered, with an effective recovery rate of 95.85%. The participants were aged 17–21 years; 1,239 were male and 2,637 were female. Chronological age was not significantly associated with GAI usage demand (*β* = −0.029, *p* = 0.071) and was therefore not entered into the multivariable model. Among the participants, 69.7% were majoring in medical-related fields, and 56.3% had a rural household registration. Regarding GAI training during high school, 9.8% of the students reported systematic provision and 41.7% reported occasional provision; thus, 51.5% had received at least some exposure, while 48.5% reported that such training had never been offered. In addition, 15.0% of the students had high-performance GAI equipment at home. A small proportion (4.2%) of the students had a thorough understanding of GAI, and almost all (98%) had not taken any GAI-related courses. 10.4% of the students were frequently exposed to GAI in professional learning, 12.9% reported that their colleges were well equipped with GAI facilities, 13.4% used GAI tools regularly, and a total of 15.7% of the students reported that their teachers frequently used GAI to support teaching in most classes.

The results of univariate analysis showed that there were statistically significant differences in gender, academic year, major, place of origin, GAI training experience in high school, family GAI equipment configuration, level of understanding of GAI, whether having taken GAI-related courses, frequency of GAI exposure in professional learning, GAI training facility conditions of the university, frequency of GAI use, and frequency of teachers’ use of GAI to assist teaching (all *p* < 0.05). However, the corresponding effect sizes were generally negligible to small (η^2^ = 0.003–0.048). The difference associated with gender was small (Cohen’s *d* = 0.238), whereas the difference associated with enrollment in GAI-related courses was moderate (Cohen’s *d* = 0.562). These findings indicate that the statistically significant group differences should be interpreted cautiously given the large sample size. Details are shown in Table 1.

**Table 1:** Comparison of GAI usage-demand scores across demographic and GAI-related characteristics (*N* = 3,876).

**Characteristics**	***n* (%)**	**GAI usage-demand score, mean ± SD**	**t/F**	***P***	**Effect size**
Gender			6.543	<0.001	*d* = 0.238
Male	1,239 (32.0)	60.29 ± 14.04			
Female	2,637 (68.0)	57.27 ± 12.00			
Academic year			9.158	<0.001	η^2^ = 0.005
First year	1,753 (45.2)	59.09 ± 13.21			
Second year	1,186 (30.6)	57.05 ± 11.95			
Third year	937 (24.2)	58.12 ± 12.81			
Major			6.995	<0.001	η^2^ = 0.005
Medicine and health sciences	2,702 (69.7)	57.85 ± 12.59			
Engineering and technology	372 (9.6)	61.08 ± 12.85			
Education and public administration	135 (3.5)	58.16 ± 12.37			
Other	667 (17.2)	58.20 ± 13.34			
Place of origin			3.712	0.011	η^2^ = 0.003
Urban	458 (11.8)	59.57 ± 13.35			
County town	756 (19.5)	58.79 ± 13.11			
Township	481 (12.4)	58.63 ± 12.04			
Rural	2,181 (56.3)	57.67 ± 12.65			
GAI training during high school			30.329	<0.001	η^2^ = 0.015
Systematically offered	379 (9.8)	62.86 ± 14.98			
Occasionally offered	1,616 (41.7)	58.22 ± 12.29			
Never offered	1,881 (48.5)	57.31 ± 12.48			
Home GAI learning equipment configuration			25.213	<0.001	η^2^ = 0.013
High-end configuration	582 (15.0)	60.99 ± 13.99			
Mid-range configuration	2,831 (73.0)	58.13 ± 12.15			
Basic configuration	463 (12.0)	55.42 ± 14.12			
Level of understanding of GAI			39.932	<0.001	η^2^ = 0.040
Excellent understanding	164 (4.2)	66.74 ± 15.71			
Good understanding	1,406 (36.3)	60.14 ± 12.39			
Moderate/basic understanding	1,699 (43.8)	56.82 ± 11.63			
Limited understanding	449 (11.6)	55.38 ± 12.18			
No understanding	158 (4.1)	55.75 ± 18.60			
Enrollment in GAI-related courses			4.885	<0.001	d = 0.562
Yes	77 (2.0)	65.25 ± 12.89			
No	3,799 (98.0)	58.09 ± 12.73			
Frequency of GAI exposure in professional learning			47.066	<0.001	η^2^ = 0.024
Frequent exposure	403 (10.4)	63.38 ± 14.45			
Occasional exposure	1,987 (51.3)	58.45 ± 11.91			
No exposure	1,486 (38.3)	56.55 ± 13.00			
Availability of GAI equipment at school			74.158	<0.001	η^2^ = 0.037
Well-equipped	499 (12.9)	64.33 ± 13.79			
Moderate	2,146 (55.4)	57.91 ± 11.68			
Inadequate	1,231 (31.8)	56.32 ± 13.39			
Frequency of active GAI use			65.636	<0.001	η^2^ = 0.048
Frequently	518 (13.4)	63.85 ± 13.65			
Occasionally	1,612 (41.6)	59.16 ± 11.69			
Rarely	1,379 (35.6)	56.21 ± 11.85			
Never	367 (9.5)	53.80 ± 15.76			
Student-reported frequency of teachers’ use of GAI in teaching			66.913	<0.001	η^2^ = 0.033
Frequent use in most classes	609 (15.7)	63.06 ± 13.53			
Occasional use in some classes	2,202 (56.8)	58.12 ± 11.65			
Rarely	1,065 (27.5)	55.69 ± 13.73			

GAI, generative artificial intelligence; SD, standard deviation. Effect sizes are reported as absolute Cohen’s *d* values for independent-samples *t*-tests and η^2^ values for one-way analyses of variance. For gender, the Welch *t*-test result is reported because the homogeneity-of-variance assumption was not met. For enrollment in GAI-related courses, the equal-variance *t*-test result is reported. Only demographic and GAI-related variables meeting the *p* < 0.05 criterion in the univariate analyses are presented. Chronological age was examined separately but did not meet this criterion (*p* = 0.071).

### 3.2 Scores of GAI needs, learning engagement, and academic resilience

Exploratory factor analysis supported a three-factor solution for the modified UWES-S. The Kaiser–Meyer–Olkin value was 0.971, Bartlett’s test of sphericity was significant, χ^2^(136) = 47,031.800, *p* < 0.001, and the three factors explained 69.372% of the total variance. The factor structure was broadly consistent with the intended dimensions of vigor, dedication, and absorption. Together with the high internal consistency coefficients, these findings provide preliminary, sample-specific support for the intended measurement structure of the modified response format. However, they should not be interpreted as a full psychometric revalidation of the UWES-S.

The results showed that: the score of GAI needs was (58.23 ± 12.77); the score of learning engagement was (53.23 ± 12.05); the score of academic resilience was (49.14 ± 9.53); the scores of each dimension are shown in Table 2.

**Table 2:** Scores of GAI usage demand, learning engagement, and academic resilience among vocational college students.

Variable/Scale	Minimum	Maximum	Score, mean ± SD
Understanding and cognition	3	15	8.46 ± 2.43
Skills and literacy	6	30	19.84 ± 4.88
Hopes and expectations	9	45	29.93 ± 7.36
GAI application status and usage demand scale	18	90	58.23 ± 12.77
Vigor	6	30	18.42 ± 4.58
Dedication	5	25	16.07 ± 3.87
Absorption	6	30	18.74 ± 4.54
Learning engagement	17	85	53.23 ± 12.05
Execution ability	5	25	16.18 ± 3.50
External support	4	20	13.44 ± 2.98
Self-efficacy	3	15	9.79 ± 2.16
Emotional stability	3	15	9.72 ± 2.12
Academic resilience	15	75	49.14 ± 9.53

### 3.3 Correlation analysis

The results of this study showed that the GAI Usage Demand Scale and its various dimensions were positively correlated with learning engagement and academic resilience. Details are shown in Table 3.

**Table 3:** Correlation analysis among GAI usage demand, learning engagement, and academic resilience.

Variable	1	2	3	4	5	6
1. Understanding and Cognition	1					
2. Skills and Literacy	0.375**	1				
3. Hopes and Expectations	0.343**	0.807**	1			
4. GAI Application Status and Usage Demand Scale	—	—	—	1		
5. Learning Engagement	0.213**	0.353**	0.383**	0.396**	1	
6. Academic Resilience	0.200**	0.371**	0.375**	0.396**	0.663**	1

Values are Pearson’s correlation coefficients. **p < 0.001. Correlations between the total GAI scale score and its constituent dimensions are not presented because they represent part–whole correlations. GAI, generative artificial intelligence.

### 3.4 Multivariate analysis

Variables with *p* < 0.05 in the univariate analyses, together with learning engagement and academic resilience, were entered simultaneously into the multiple linear regression model, resulting in a total of 14 candidate independent variables. The total GAI usage demand score was used as the dependent variable. The results indicated that gender, academic year, family GAI equipment configuration, level of understanding of GAI, whether having taken GAI-related courses, frequency of GAI use, frequency of teachers’ use of GAI for teaching assistance, learning engagement, and academic resilience were independently associated with GAI usage demand among vocational college students (all *p* < 0.05). The overall regression model was statistically significant, *F*(14, 3,861) = 88.932, *p* < 0.001, with *R*^2^ = 0.244 and adjusted *R*^2^ = 0.241. Five variables that were statistically significant in the univariate analyses—major, place of origin, GAI training during high school, frequency of GAI exposure in professional learning, and availability of GAI equipment at school—were no longer statistically significant after multivariable adjustment. Notably, although the availability of GAI equipment at school showed a comparatively larger univariate difference (*F* = 74.158, η^2^ = 0.037), its adjusted association did not reach statistical significance (*p* = 0.093). This attenuation indicates that the unadjusted group differences may partly reflect variation shared with other variables included in the model. Details of the multiple linear regression analysis are shown in Table 4.

**Table 4:** Multiple linear regression analysis of variables associated with GAI usage demand.

**Variables**	***B***	**SE**	***β***	***t***	***P***	**95% CI for *B***	**VIF**
Constant	54.539	3.004	—	18.155	<0.001	[48.649, 60.429]	—
Academic year	−0.764	0.229	−0.048	−3.340	0.001	[−1.212, −0.316]	1.065
Gender	−2.750	0.392	−0.100	−7.006	<0.001	[−3.519, −1.980]	1.050
Place of origin	−0.280	0.168	−0.024	−1.663	0.096	[−0.610, 0.050]	1.079
Home GAI learning equipment configuration	−1.120	0.375	−0.045	−2.984	0.003	[−1.856, −0.384]	1.187
Enrollment in GAI-related courses	−2.809	1.310	−0.031	−2.144	0.032	[−5.378, −0.241]	1.047
Level of understanding of GAI	−0.897	0.242	−0.061	−3.702	<0.001	[−1.372, −0.422]	1.376
Frequency of active GAI use	−1.874	0.255	−0.123	−7.357	<0.001	[−2.373, −1.375]	1.421
Student-reported frequency of teachers’ use of GAI in teaching	−1.170	0.317	−0.059	−3.685	<0.001	[−1.792, −0.547]	1.320
Frequency of GAI exposure in professional learning	0.353	0.342	0.018	1.032	0.302	[−0.317, 1.023]	1.498
Availability of GAI equipment at school	−0.570	0.339	−0.029	−1.680	0.093	[−1.235, 0.095]	1.482
Major	0.024	0.160	0.002	0.152	0.880	[−0.290, 0.339]	1.064
Learning engagement	0.217	0.020	0.205	10.808	<0.001	[0.177, 0.256]	1.829
Academic resilience	0.316	0.025	0.236	12.581	<0.001	[0.267, 0.365]	1.794
GAI training during high school	0.478	0.303	0.025	1.576	0.115	[−0.117, 1.073]	1.248

B, unstandardized regression coefficient; SE, standard error; β, standardized regression coefficient; CI, confidence interval; VIF, variance inflation factor. The total GAI usage-demand score was entered as the dependent variable. All 14 candidate independent variables were entered simultaneously using the Enter method. Model summary: *R*^2^ = 0.244, adjusted *R*^2^ = 0.241, *F*(14, 3,861) = 88.932, *p* < 0.001. Coding of categorical and ordinal predictors: academic year, 1 = first year, 2 = second year, 3 = third year; gender, 1 = male, 2 = female; place of origin, 1 = urban, 2 = county town, 3 = township, 4 = rural; home GAI learning equipment configuration, 1 = high-end, 2 = mid-range, 3 = basic; enrollment in GAI-related courses, 1 = yes, 2 = no; level of understanding of GAI, 1 = excellent, 2 = good, 3 = moderate/basic, 4 = limited, 5 = none; frequency of active GAI use, 1 = frequently, 2 = occasionally, 3 = rarely, 4 = never; student-reported frequency of teachers’ use of GAI in teaching, 1 = frequent use in most classes, 2 = occasional use in some classes, 3 = rarely; frequency of GAI exposure in professional learning, 1 = frequent, 2 = occasional, 3 = none; availability of GAI equipment at school, 1 = well-equipped, 2 = moderate, 3 = inadequate; major, 1 = medicine and health sciences, 2 = engineering and technology, 3 = education and public administration, 4 = other; GAI training during high school, 1 = systematically offered, 2 = occasionally offered, 3 = never offered. Learning engagement and academic resilience were entered as total scores.

To examine potential criterion contamination, a sensitivity analysis was conducted after excluding the three items of the Understanding and Cognition dimension from the dependent variable. The resulting 15-item score ranged from 15 to 75 (49.77 ± 11.66). The sensitivity model was statistically significant, *F*(14, 3,861) = 86.475, *p* < 0.001, with *R*^2^ = 0.239 and adjusted *R*^2^ = 0.236. Level of understanding of GAI remained independently associated with the reduced GAI usage-demand score (*B* = −0.814, SE = 0.222, *β* = −0.060, *t* = −3.668, *p* < 0.001, 95% CI [−1.249, −0.379]; VIF = 1.376). This finding suggests that the observed association was not solely attributable to the inclusion of the potentially overlapping Understanding and Cognition dimension.

## 4 Discussion

### 4.1 Current status of GAI usage demand among vocational college students

The mean total GAI usage-demand score was 58.23 ± 12.77, which was slightly above the theoretical midpoint of 54 for the 18-item scale, with a possible score range of 18–90. Because validated normative cutoff values are unavailable, the observed score was not categorized as indicating a low, moderate, or high level of GAI usage demand. At the dimension level, the per-item mean scores were 3.326 ± 0.817 for Hopes and Expectations, 3.307 ± 0.814 for Skills and Literacy, and 2.820 ± 0.810 for Understanding and Cognition. The scores for Hopes and Expectations and Skills and Literacy were similar in magnitude, differing by only 0.019, whereas Understanding and Cognition had a descriptively lower score. Because no inferential comparisons among the dimension scores were conducted, these differences should be interpreted descriptively rather than as statistically significant differences.

The total GAI usage-demand score in the present sample was slightly lower than that reported by Hu et al. (2025). This discrepancy may reflect differences in sample characteristics and institutional contexts. All participants in the present study were vocational college students, whose technology exposure, basic information literacy, and disciplinary application contexts may differ from those of students attending general undergraduate or comprehensive universities. Differences in GAI infrastructure and the extent to which GAI is integrated into teaching across regions and types of institutions may also be relevant.

These descriptive findings suggest that future needs assessments should consider all three dimensions rather than prioritize educational initiatives solely according to their rank order. Higher education institutions may consider balanced GAI literacy initiatives addressing foundational knowledge, ethical boundaries, practical application skills, and critical evaluation of GAI-generated content. However, the present study did not evaluate the effectiveness of such initiatives; these suggestions should therefore be regarded as considerations for future educational development rather than as interventions supported by the current findings (Wu et al., 2023; Wang et al., 2025).

### 4.2 Factors associated with GAI demand among vocational college students

#### 4.2.1 General information

After multivariable adjustment, gender, academic year, availability of home GAI learning equipment, level of understanding of GAI, enrollment in GAI-related courses, frequency of active GAI use, and students’ reported frequency of their teachers’ use of GAI in teaching were independently associated with GAI usage demand. These findings indicate that students’ demand for GAI was associated with a combination of individual, educational, and technology-related characteristics. However, given the cross-sectional design, these variables should be interpreted as correlates rather than causal determinants of GAI usage demand.

Male students reported higher GAI usage demand than female students, while students in earlier academic years reported higher demand than those in later academic years. However, the present study did not assess technological self-efficacy, exploratory interest, technology anxiety, or adaptation to academic demands. Therefore, the psychological mechanisms underlying the observed gender- and academic-year-related differences cannot be determined. These findings should not be interpreted as indicating that either female students or students in later academic years have technological deficits. Future studies should examine whether the observed differences can be explained by technology-related experience, self-efficacy, learning opportunities, or other contextual variables.

The availability of home GAI learning equipment was positively associated with GAI usage demand, suggesting that access to relevant devices may be an important contextual correlate of students’ reported demand. Level of GAI understanding and enrollment in GAI-related courses were also positively associated with GAI usage demand. These findings suggest that students’ familiarity with GAI and their exposure to structured learning opportunities may be relevant when assessing their educational needs. This interpretation is broadly consistent with technology-acceptance research emphasizing the relevance of users’ technology-related perceptions to technology adoption intentions (Davis, 1989). Nevertheless, the present study did not measure perceived usefulness, perceived ease of use, satisfaction, or habitual use; therefore, these constructs cannot be regarded as mechanisms established by the present analysis.

Frequency of active GAI use and students’ reported frequency of their teachers’ use of GAI in teaching were also positively associated with students’ GAI usage demand. Previous empirical studies have reported associations of student–GAI interaction and GAI acceptance with cognitive engagement, self-efficacy, and overall learning engagement (Liang et al., 2023; Deng et al., 2026). However, the temporal direction of the associations observed in the present study remains uncertain. More frequent active use of GAI and greater student-reported exposure to teachers’ use of GAI may be associated with stronger demand; conversely, students with stronger pre-existing demand may be more likely to use GAI actively and pay attention to its use by teachers. Longitudinal studies are needed to clarify these relationships.

From a practical perspective, educational institutions may consider assessing students’ access to basic GAI learning resources, providing structured GAI literacy education, and supporting teachers in the critical and responsible integration of GAI into teaching (Mallik and Gangopadhyay, 2023). However, although the availability of GAI equipment at school was associated with GAI usage demand in the univariate analysis, this association was no longer statistically significant after multivariable adjustment. Therefore, the present study does not establish an independent association between institutional equipment availability and students’ GAI usage demand and does not, by itself, provide sufficient evidence that infrastructure expansion would increase such demand. Decisions regarding institutional resource investment should be informed by further needs assessment and prospective evaluation. Educational initiatives should support students with different levels of prior experience and access rather than presume deficits on the basis of gender or academic year. They should also emphasize the functional boundaries, ethical risks, and critical evaluation of GAI-generated content. Because the present study did not evaluate the effects of these strategies, they should be regarded as considerations for future educational development rather than interventions demonstrated to be effective by the current findings.

#### 4.2.2 Learning engagement

This study found a significant positive correlation between vocational college students’ GAI usage demand and learning engagement. Huang et al. (2023) investigated the effects of AI-enabled personalized video recommendations in a flipped classroom and found a significant improvement in online learning engagement among students with moderate motivation, whereas comparable effects were not observed among students with high or low motivation. Their findings suggest that the engagement-related effects of AI-supported learning activities may vary according to students’ motivational characteristics. Learning engagement refers to the sustained positive psychological and behavioral states exhibited by students in learning activities, and its core dimensions include vigor, dedication, and absorption (Fredricks et al., 2004; Schaufeli et al., 2002). Higher learning engagement has been associated with stronger intrinsic motivation, deeper cognitive participation, greater persistence (Lam et al., 2012), and better academic performance (Carini et al., 2006). From a theoretical perspective, metacognition—the capacity to monitor and regulate one’s thinking and learning processes—has been regarded as an important basis for self-regulated and lifelong learning (Wijnen-Meijer, 2020). However, metacognition was not measured in the present study and is therefore not interpreted as an empirically established correlate of learning engagement in this sample.

Based on this, this study argues that students with high learning engagement are more likely to perceive GAI as an advanced cognitive auxiliary tool, and proactively utilize it for knowledge exploration, content generation, and problem-solving, thereby demonstrating stronger usage willingness and functional demands. Conversely, functions provided by GAI—such as real-time feedback, personalized information integration, and creative content generation—may also support and enhance students’ cognitive engagement and behavioral participation to a certain extent by reducing cognitive load and expanding thinking boundaries. Therefore, there may be a bidirectional promotion mechanism between the two, rather than a one-way causal relationship. It should be emphasized that due to the cross-sectional study design, this research cannot determine the temporal sequence and causal direction between variables. The above interpretations are merely reasonable inferences based on existing theories, and future studies urgently need to verify them through longitudinal tracking or experimental intervention research.

Furthermore, when promoting the educational application of GAI in colleges and universities today, the focus should not be solely on the technology itself, but rather on the coupling mechanism between GAI and students’ deep learning psychology. For instance, GAI can be leveraged to design challenging and open-ended inquiry tasks that stimulate students’ cognitive curiosity and intrinsic motivation for independent exploration; alternatively, its interactive features can be utilized to create a supportive learning environment, enhancing students’ concentration and learning engagement (Mallik and Gangopadhyay, 2023). In this process, teachers’ role should shift from “technology promoters” to “learning facilitators,” helping students effectively transform high learning engagement into the critical and purposeful use of GAI tools, while avoiding superficial dependence or information overload.

In summary, higher learning engagement was associated with greater GAI usage demand. However, the cross-sectional design does not establish the temporal or causal direction of this association. Longitudinal and experimental studies are needed to determine whether learning engagement contributes to GAI usage demand, whether GAI use subsequently supports learning engagement, or whether the relationship is bidirectional.

#### 4.2.3 Academic resilience

This study found that academic resilience was positively associated with GAI usage demand among vocational college students. Academic resilience refers to an individual’s ability to effectively adapt and maintain or restore positive learning functioning when facing academic stress, setbacks, or adversities (Martin and Marsh, 2008). Daily academic resilience emphasizes students’ adaptive strategies for coping with challenges in routine learning contexts, such as recovering from failure, adjusting learning methods, seeking internal and external resources, and transforming negative experiences into opportunities for growth (Martin and Marsh, 2020). Students with greater academic resilience generally demonstrate stronger autonomy and a more proactive problem-solving orientation.

One possible interpretation of the observed association is that students with greater academic resilience may be more likely to perceive GAI as a potentially useful learning-support resource. When encountering knowledge gaps or complex learning tasks, these students may be more inclined to seek immediate explanations, alternative problem-solving approaches, or structured knowledge support through GAI. However, these behavioral processes were not directly measured in the present study and should therefore be regarded as theoretical interpretations rather than empirically tested mechanisms.

Therefore, colleges and universities can organically integrate academic resilience training with GAI literacy education. Teachers should design moderately challenging tasks in teaching and guide students to consciously use GAI as a tool for auxiliary analysis, knowledge reconstruction, and strategy generation when encountering difficulties, rather than as a direct provider of answers. However, the cross-sectional design does not establish the temporal or causal direction of the association between academic resilience and GAI usage demand. Furthermore, the present study did not evaluate whether GAI-based educational activities improve students’ problem-solving abilities, self-efficacy, or academic resilience. These potential relationships and effects should be examined in future longitudinal and intervention studies.

## 5 Conclusion

This study found that vocational college students’ GAI usage demand was slightly above the theoretical midpoint of the scale and was associated with several individual, educational, technological, and psychological characteristics, including learning engagement and academic resilience. These findings may help educational institutions identify areas for further assessment when developing GAI literacy initiatives. Given the cross-sectional design, the observed associations should not be interpreted as evidence of causal relationships. Longitudinal and intervention studies are needed before specific causal or educational effects can be established.

### 5.1 Limitations

This study has several limitations. First, the cross-sectional design makes it difficult to infer causality and temporal dynamics, and longitudinal or intervention studies should be adopted in the future; Second, the sample is limited to vocational colleges in Sichuan Province, resulting in restricted external validity, and cross-regional comparative studies need to be carried out; Third, the model’s explanatory power is relatively low (adjusted *R*^2^ = 0.241), indicating that potential variables such as school support, peer influence, and technology anxiety, should be examined in future research. Fourth, candidate variables for the regression model were selected partly according to statistical significance in the univariate analyses. This data-driven screening approach may have introduced model-selection bias and may have excluded variables with limited univariate associations but potential relevance after multivariable adjustment. Future studies should prespecify candidate predictors on the basis of an explicit theoretical framework and validate the resulting model in an independent sample. Fifth, although participants were recruited from five vocational colleges, participant-level institutional identifiers were unavailable, preventing adjustment for within-college clustering. Consequently, standard errors may have been underestimated and confidence intervals may have been too narrow. Future multicenter studies should retain institutional identifiers and use multilevel or cluster-robust methods to account for the hierarchical sampling structure. Sixth, measurement-related limitations should be considered. The Daily Academic Resilience Scale was originally developed for middle-school students, and two subscales showed modest internal consistency in the present sample (*α* = 0.633–0.653); meanwhile, the UWES-S response format was modified from the original 7-point to a 5-point scale without full independent psychometric validation. These issues may affect measurement precision, and future studies should further validate both instruments in vocational college populations.

## Data Availability

The data that support the findings of this study are available from the corresponding author upon reasonable request.
